# Therapeutic Effect of Teneligliptin in Drug-Induced Nephrotoxicity: An In-Vitro Study

**DOI:** 10.7759/cureus.23871

**Published:** 2022-04-06

**Authors:** Tülay Becerir, Onur Tokgün, Kubilay İnci, İlknur Girişgen, Selcuk Yuksel

**Affiliations:** 1 Department of Pediatric Nephrology, Pamukkale University School of Medicine, Denizli, TUR; 2 Department of Medical Genetics, Pamukkale University School of Medicine, Denizli, TUR; 3 Department of Cancer Molecular Biology, Institute of Medical Sciences, Pamukkale University, Denizli, TUR; 4 Department of Pediatric Nephrology, Pamukkale University, Faculty of Medicine, Denizli, TUR; 5 Department of Pediatric Rheumatology and Pediatric Nephrology, Pamukkale University School of Medicine, Denizli, TUR

**Keywords:** gentamicin, vancomycin, cisplatin, drug-induced nephrotoxicity, teneligliptin

## Abstract

Background

Drug-induced nephrotoxicity is an important side effect of many commonly used drugs. In this study, we planned to evaluate the effects of teneligliptin (TG), which is a dipeptidyl peptidase-4 (DPP-4) inhibitor, on cell healing by creating nephrotoxicity models in human renal proximal tubule cell and human embryonic kidney epithelial cells cell lines in-vitro with cisplatin, vancomycin, and gentamicin.

Methodology

First, we determined the 50% inhibitory concentration doses of nephrotoxic drugs and the nephroprotective dose of TG. Then, we analyzed the difference in cell viability, apoptosis, and oxidative stress (reactive oxygen and nitrogen species (ROS/RNS) production) between TG-treated and untreated cells after nephrotoxicity occurred. Moreover, we evaluated the expression of kidney injury molecule-1 (KIM-1) and neutrophil gelatinase-associated lipocalin (NGAL) in cells.

Results

We found that when cell lines were treated after toxicity was induced with TG, cell viability increased, apoptosis and ROS/RNS production were significantly decreased, and expressions of KIM-1 and NGAL were significantly reduced.

Conclusions

This study showed that TG has positive effects on the recovery of drug-induced nephrotoxicity in an in-vitro setting.

## Introduction

Drug-induced nephrotoxicity is an important side effect of many drugs commonly used in clinical practice, which prevents the effective use of many drugs (chemotherapeutics, antibiotics, etc.) and causes serious damage to the kidney. Preventing drug-induced nephrotoxicity enables patients to receive effective treatment and reduces mortality and morbidity due to acute kidney injury (AKI) [1,2]. The general approach in the treatment of drug-induced nephrotoxicity is the administration of the drug at the lowest possible dose and duration, fluid therapy, and increasing the urine output. However, the effectiveness of these treatments is insufficient in preventing and treating the development of nephrotoxicity [1].

Teneligliptin (TG) is a recently developed oral dipeptidyl peptidase-4 (DPP-4) inhibitor. DPP-4 inhibitors are used for glycemic regulation in patients with type 2 diabetes. These inhibitors increase insulin secretion and improve insulin resistance [3]. However, DPP-4 inhibitors exert nephroprotective effects that are independent of their blood sugar-controlling effects. The curative effects of these inhibitors on diabetic nephropathy have been reported [4]. In studies conducted on rats, they have been shown to alleviate AKI induced by cisplatin as well as by ischemia-reperfusion injury [5,6].

In this study, we aimed to evaluate the therapeutic effects of TG on drug-induced nephrotoxicity. For this, we planned to evaluate the effect of TG on healing by inducing nephrotoxicity in an in-vitro setting in human renal proximal tubular cells (PTCs) (HK-2) and human embryonic kidney epithelial cells (HEK293T) cell lines using nephrotoxic drugs such as cisplatin (Cis), vancomycin (Van), and gentamicin (Gen).

## Materials and methods

Cell culture

HK-2 was cultured in T75 flasks in Dulbecco’s modified eagle medium (DMEM)/nutrient mixture F-12 (Gibco, NY, USA) medium by adding 10% fetal bovine serum (FBS) at 37°C in 5% CO_2_ and 95% humidity. HEK293T cell line was cultured in T75 flasks in DMEM (Gibco, NY, USA) medium with 10% FBS at 37°C in 5% CO_2_ and 95% humidity.

Determination of 50% inhibitory concentration doses of nephrotoxic drugs

After the proper proliferation dynamics of HEK293T and HK-2 cell lines were achieved, cells were seeded into 96-well plates at 1 × 10^4^ cells/well (100 mL). By changing the medium 24 hours after the cells were cultivated, Cis (Toronto Research Chemical, Toronto, Canada) at doses of 1 µM, 10 µM, 50 µM, 100 µM, and 200 µM; Van (Vankomax®) at doses of 10 µM, 100 µM, 1,000 µM, 2,000 µM, 4,000 µM, and 5,000 µM; and Gen at doses of 1,000 µM, 5,000 µM, 10,000 µM, and 20,000 µM were applied. The cells were then cultured for 48 hours at 37°C in 5% CO^2^ and at 95% humidity. At the end of the incubation period, cell viability in the wells was analyzed using the cell counting kit-8 (CCK-8) (Abbkine, California, USA). Following the kit protocol, 5 µL of kit chemical was added to the wells after 48 hours of incubation. At the end of incubation at 37°C for three hours, absorbance values were measured at 450 nm using the GloMax® device (Promega, Madison, WI, USA).

Determination of the nephroprotective dose of teneligliptin

Cells were seeded onto 96-well plates at 1 × 10^4^ cells/well (100 mL). For TG doses of 1, 10, 100, and 1,000 nM, 50% inhibitory concentration (IC50) doses of a nephrotoxic agent were applied simultaneously 24 hours after the cells were seeded, with each dose applied three times. At the end of a total of 48 hours of incubation, cellular viability was analyzed according to the CCK-8 kit protocol.

Apoptosis assay

The TUNEL chromogenic apoptosis detection kit (ABP Bioscience, Beltsville, MD, USA) was used for apoptosis analysis. HEK293T and HK-2 cell lines were inoculated into 24-well plates at 2.5 × 10^4^ cells/well. Cells were treated with 0.05% trypsin-0.53 mM ethylenediaminetetraacetic acid (EDTA) (for HK-2 cell line) and 0.25% trypsin-0.53v mM EDTA (for HEK293T cell line) and transferred into 1.5 mL Eppendorf cell culture flasks. Living and non-living cells were precipitated by centrifugation following the kit protocol. Then, cell pellets fixed with formaldehyde and triton-x-100 were dropped onto a slide and pre-prepared using incubation at 37°C. Double-stranded DNA fragments in apoptotic cells in the prepared samples were passed through the kit solutions and labeled with biotin-11-dUTP using the terminal deoxynucleotidyl transferase (TdT) enzyme. Marked cells were stained with methyl green dye, and dark-colored apoptotic cells were then analyzed under a light microscope.

Oxidative stress analysis

Intracellular reactive oxygen and nitrogen species (ROS and RNS) levels were evaluated using the Cellular ROS/RNS Detection Assay Kit (Abcam, USA; catalog number: ab139473) according to the manufacturer’s protocol. Briefly, cells were plated on 24 wells, and following the drug treatments, the cells were captured under a fluorescence microscope at 490-650 nm. The fluorescence intensity was measured using the ImageJ software.

Analysis of kidney injury molecule-1 and neutrophil gelatinase-associated lipocalin

After incubation with nephrotoxic agents, TG-treated and untreated cells were removed from their media in a vacuum, placed on ice, collected with 500 µL Trizol (Qiazol, Qiagen, Hilden, Germany), and then placed in 1.5 mL Eppendorf flasks. RNA isolations were performed. Total RNA samples were translated into cDNA using the Hi-Capacity cDNA Reverse Transcription Kit (Applied Biosystems, Foster City, CA, USA). To analyze the expressions of biomarkers including kidney injury molecule-1 (KIM-1) and neutrophil gelatinase-associated lipocalin (NGAL) on cDNA samples, a quantitative real-time polymerase chain reaction (qRT-PCR) reaction was performed in Bio-Rad CFX-96 device using 2X SYBR green (Quantitech, Qiagen, Hilden, Germany) master mix. Before preparing the reaction mixture, cDNA samples were diluted at a ratio of 1:5 using nuclease-free water. Then, the reaction mixture was prepared with 2 μL of cDNA sample, 10 μL of 2X master mix, 0.3 μM forward and reverse primers, with a total volume of 20 μL. Glyceraldehyde 3-phosphate dehydrogenase (GAPDH) was used as an endogenous control to analyze the results. The reaction cycle was performed in three repetitions for each sample.

Statistical analysis

Graphpad Prism software (version 8.0.1) was used for the statistical analysis of the results. One-way analysis of variance (ANOVA) and Tukey’s test were applied to compare the untreated control against the treated groups. Statistical significance was set at p-values of <0.05.

## Results

IC50 doses and nephroprotective dose of teneligliptin

**Figure 1:**
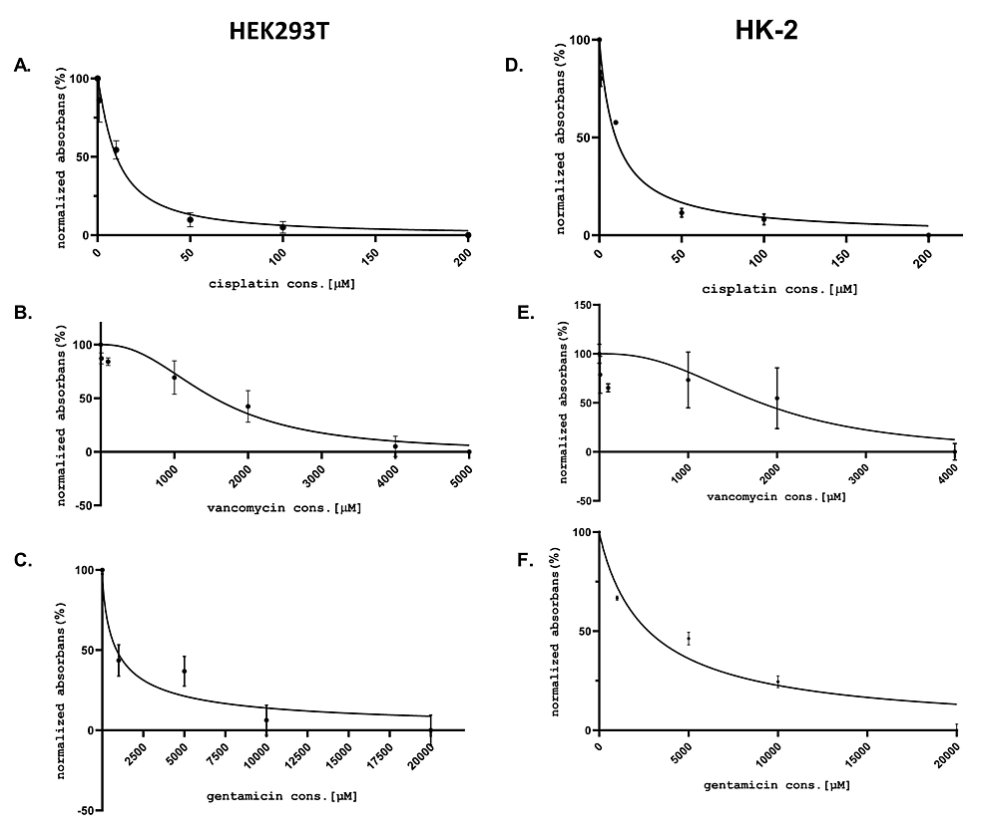
The effects of cisplatin, vancomycin, and gentamicin on cellular toxicity in HEK293T and HK-2 cell lines. IC50 doses of nephrotoxic drugs (cisplatin, vancomycin, and gentamicin). IC50: 50% inhibitory concentration

**Figure 2:**
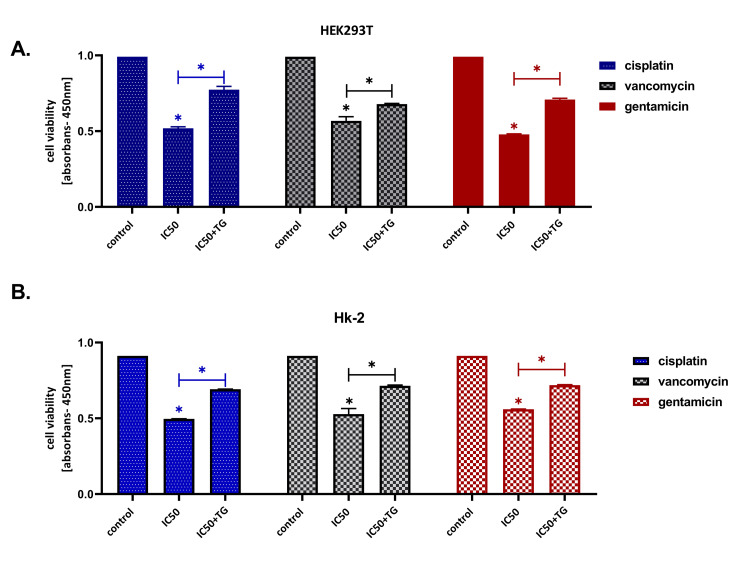
Cell viability measurements in the evaluation of the therapeutic effect of teneligliptin in HK-2 (A) and HEK293T (B) cell lines. Teneligliptin significantly increases cell viability after the development of drug-induced nephrotoxicity in HK-2 and HEK293T cell lines. *P < 0.05; Cis: cisplatin; Van: vancomycin; Gen: gentamicin; IC50: 50% inhibitory concentration

**Figure 3:**
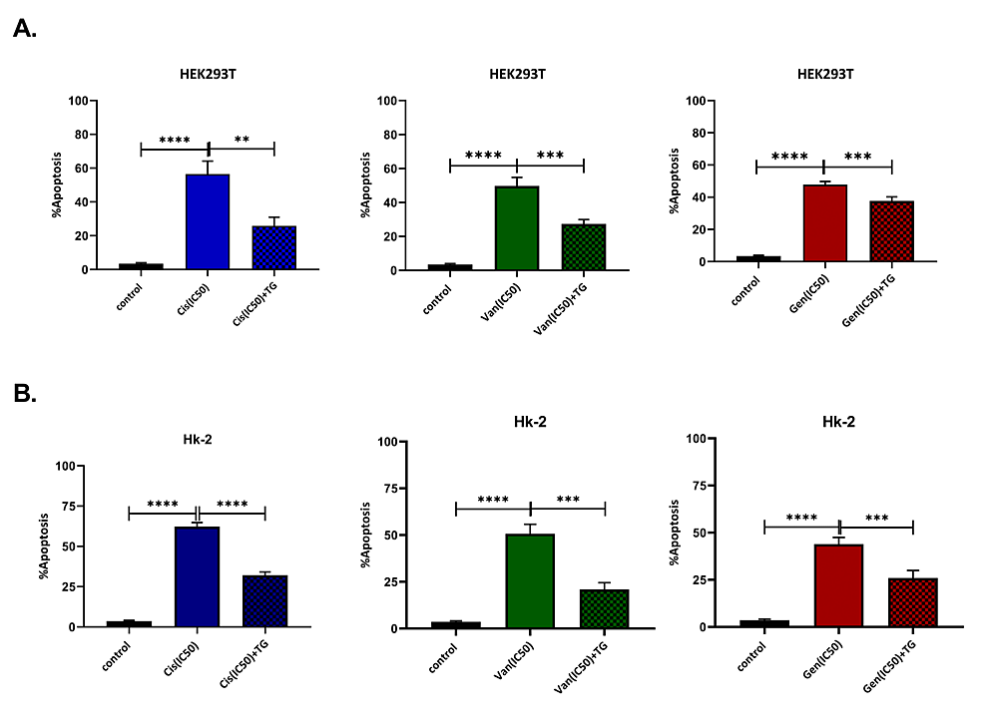
Analysis results of the antiapoptotic effect of teneligliptin in HK-2 (A) and HEK293T (B) cell lines. After the development of nephrotoxicity, teneligliptin treatment reduces the number of apoptotic cells. **P < 0.01; ***P < 0.001; ****P < 0.0001; Cis: cisplatin; Van: vancomycin; Gen: gentamicin; TG: teneligliptin; IC50: 50% inhibitory concentration

The IC50 doses determined separately for both cell cultures of Cis, Van, and Gen are given in Figure 1. The nephroprotective dose of TG was determined as 100 nM, and this dose was used in the later stages of the experiment. TG increased cell viability and reduced apoptosis after the development of drug-induced nephrotoxicity HK-2 and HEK293T cell lines were treated with all three drugs for 24 hours to evaluate the therapeutic effect of TG after the development of nephrotoxicity. In the previous stage of the study, it was observed that the cell viability decreased, and the signs of toxicity began to develop at IC50 doses at the 24th hour of the incubation. At the 24th hour of incubation, TG was added to the cell culture medium. Cell lines treated and untreated with TG were compared at 48 hours. It was observed that the cell viability increased (p < 0.05), and apoptosis decreased (p < 0.01) in cells treated with TG. Cell viability and apoptosis measurement results for all three drugs. Both cell lines are shown in Figure 2 and Figure 3.

After the development of drug-induced nephrotoxicity, TG reduces oxidative stress

**Figure 4:**
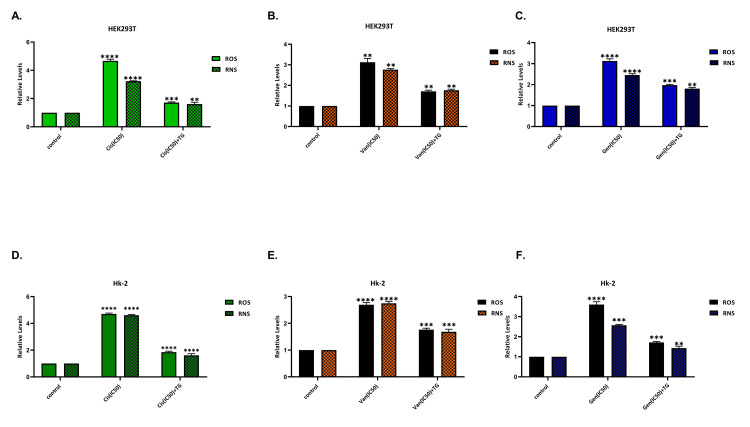
In HK-2 (A) and HEK293T (B) cell lines. Results of reactive oxygen and nitrogen species (ROS and RNS) analysis. ROS and RNS production were higher in cells that were not treated with teneligliptin when compared with those treated with teneligliptin after the development of nephrotoxicity. **P < 0.01; ***P < 0.001; ****P < 0.0001; Cis: cisplatin; Van: vancomycin; Gen: gentamicin; TG: teneligliptin

Production of ROS and RNS increased in both cell lines after the treatment with nephrotoxic agents. ROS and RNS production were higher in cells that were not treated with TG when compared with those treated with TG after the development of nephrotoxicity (p < 0.01). Results of ROS and RNS are given in Figure 4.

TG decreases the expressions of KIM-1 and NGAL in drug-induced nephrotoxicity

**Figure 5:**
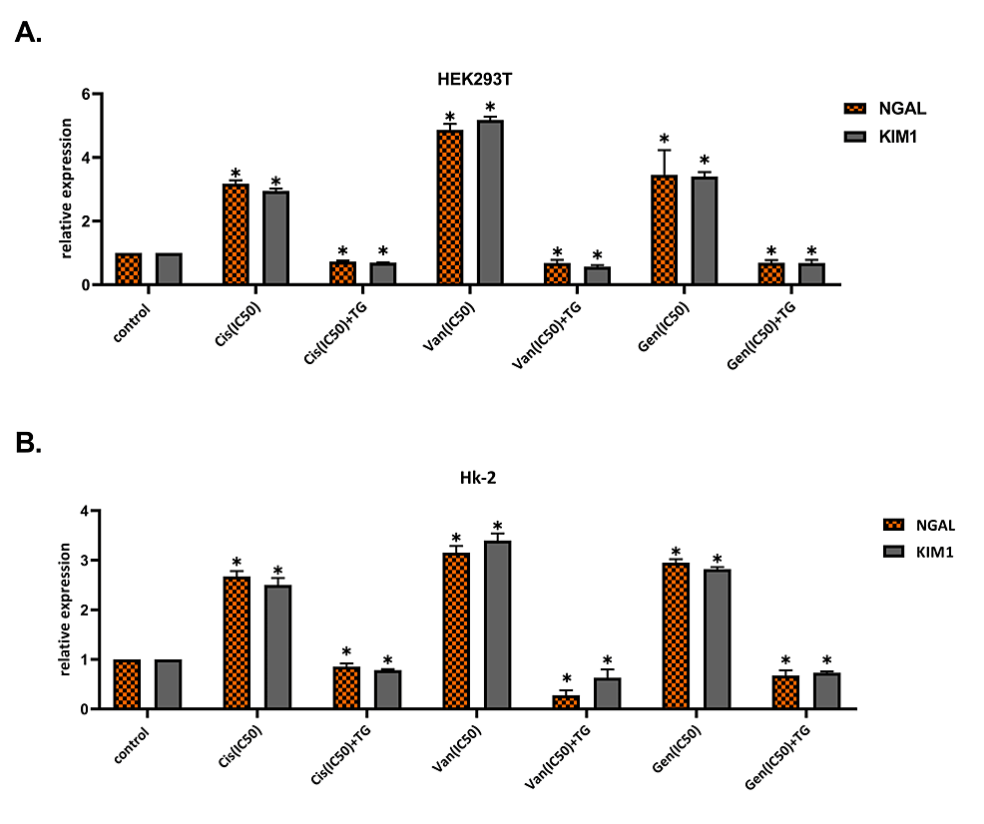
Results of analysis of kidney injury molecule-1 (KIM-1), neutrophil gelatinase-associated lipocalin (NGAL) in HEK293T (A) and HK-2 (B) cell lines. KIM-1 and NGAL expressions increased in cell lines with cisplatin, vancomycin, and gentamicin treatment. A reduction in the expression of these biomarkers in cell lines was observed when treated with teneligliptin after exposure to nephrotoxic agents. *P < 0.05; **P < 0.01; ***P < 0.001; ****P < 0.0001; Cis: cisplatin; Van: vancomycin; Gen: gentamicin; TG: teneligliptin

The expressions of KIM-1 and NGAL in cell lines increased with treatment with nephrotoxic agents. A significant reduction in the expression of these biomarkers was observed in cell lines when treated with TG after exposure to nephrotoxic agents (p < 0.01). The data concerning the expressions of KIM-1 and NGAL are given in Figure 5.

## Discussion

In this study, we aimed to evaluate the effects of TG on the recovery of drug-related nephrotoxicity. The results of our in vitro study showed that after toxicity develops, TG exerts a healing effect on cells; by increasing cell viability, and by reducing apoptosis and oxidative stress. In this study, we also showed that the expressions of KIM-1 and NGAL increased when nephrotoxicity occurred, and their expressions decreased after the treatment with teneligliptin.

Immortal cell lines are the most widely used tools to investigate the mechanism of nephrotoxicity due to their easy accessibility and easy manipulation of cells. Compared to animal models, two-dimensional (2D) cell models have much less ethical responsibility [7]. More than 10 renal cell types, including podocyte cells, proximal tubule cells, distal tubule cells, collecting duct cells, etc., are being used in nephrotoxicity studies [8]. PTCs are the most sensitive cells to drug toxicity due to their high metabolic activity in the kidney, so PTCs are often used in the evaluation of drug-induced nephrotoxicity in 2D cell lines [7,8]. In this study, we used HK-2 and HEK293T cell lines.

Teneligliptin is an oral DPP-4 inhibitor. In various studies, DPP-4 inhibitors have been shown to exert nephroprotective effects independent of their blood sugar-controlling effects and have curative effects on diabetic nephropathy [3,4]. Iwakura et al. [9] evaluated diabetic cancer patients using DDP-4 inhibitors and reported that cis-induced AKI developed less frequently in these patients. Although the mechanism of the renoprotective effect of DPP-4 is not fully known, it has been suggested that DPP-4 inhibitors provide endothelial protection by reducing oxidative stress and inflammation in clinical studies performed among patients with chronic kidney disease [10,11].

In two studies performed on rats in in-vitro settings, TG had a positive effect on cell healing by reducing inflammation and increasing tubular regeneration in cis-induced nephrotoxicity [5,12]. It has also been reported that TG has a mitogenic effect on the CXC chemokine receptor (CXCR)-4 and chemokine ligand (CXCL)-12 axis by increasing cellular proliferation and prolonging the viability of PTCs [5]. In the present study, unlike the literature, the effect of TG on Van and Gen nephrotoxicity was evaluated in drug-induced nephrotoxicity experiments. To our knowledge, this is the first study to evaluate the effect of TG on the nephrotoxic effect of Van and Gen.

Cis is an effective chemotherapeutic agent used in the treatment of various cancers such as sarcoma and lymphoma. However, its nephrotoxic side effect limits the effective use of this drug. Cis is freely filtered by the glomeruli and accumulates in renal tubular cells. Cis concentration is approximately five times higher in tubular epithelial cells than in blood [13]. At the same time, Cis can be metabolized in PTCs and cause nephrotoxicity with its metabolites [14]. Cis and its metabolites mediate cytotoxic damage by binding to DNA. DNA damages caused by Cis can trigger cell apoptosis and necrosis [13]. In addition to direct apoptosis and necrosis of tubular cells, inflammation has also been reported in the pathogenesis of Cis-induced nephrotoxicity [15]. Aminoglycosides (AMGs) are widely used clinically to treat infections caused by gram-negative bacteria. AMGs exert their nephrotoxic effects through renal tubular obstruction, renal vasoconstriction, and mesangial contraction. Among them, tubular cytotoxicity is the primary mechanism responsible for AMG-induced nephrotoxicity [16,17]. In animal studies or experiments with cell cultures, treatment with Gen or another AMG has been reported to cause apoptosis and necrosis of these cells [1,18]. Van is a widely used glycopeptide antibiotic. The exact mechanism of Van-induced nephrotoxicity has not yet been fully defined. Previously, it was reported that the initiator of nephrotoxicity was associated with oxidative stress caused by Van. Van can indirectly generate ROS and induce inflammatory events in the proximal tubules after vancomycin is reabsorbed into tubular cells [1,2]. It has also been reported that Van causes nephrotoxicity by causing the development of tubular casts [19].

In our study, we found that these three nephrotoxic drugs had a toxic effect on PTC by decreasing cell viability, increasing apoptosis, or causing oxidative damage in both cell lines. These findings were similar to those indicated in the literature. In addition, we found that when TG was added to the medium after the development of cytotoxicity, the rate of apoptosis decreased, cell viability was better, and ROS/RNS production decreased compared to cells that were not treated with TG. This finding was similar to that reported by Iwakura et al. [5] which demonstrated the positive effect of TG on the regeneration of PTCs in cis toxicity. However, we found that TG also had a positive effect in cases with Van and Gen toxicity.

In studies conducted with the current PTC culture, expressions of biomarkers are also evaluated to detect drug toxicity [20-22]. For this purpose, in our study, we evaluated the expressions of KIM-1 and NGAL. We found that expressions of KIM-1 and NGAL increased when Cis, Van, and Gen were administered alone and that the expressions of these biomarkers decreased after the treatment with TG. The decrease in the expressions of KIM-1 and NGAL with the addition of TG to the medium has confirmed the positive effects of TG on drug-related toxicity using biomarkers.

Limitations

This experimental study has all the limitations of cytotoxicity studies with 2D cell culture.

## Conclusions

In this study, we found that Cis, Van, and Gen had a toxic effect on PTC lines by decreasing cell viability and increasing apoptosis or oxidative damage in both cell lines. When cell lines were treated with TG after toxicity was induced cell viability increased, apoptosis and ROS/RNS production were significantly decreased, and expressions of KIM-1 and NGAL were significantly reduced.

The positive effects of TG on recovery from drug-induced nephrotoxicity have been demonstrated in an in-vitro setting. However, this finding needs to be supported by clinical studies. This study sheds light on future drug-induced nephrotoxicity studies.
